# Pressure-strain product reflects left ventricular stroke work under a wide range of left ventricular assist device support levels

**DOI:** 10.3389/fcvm.2025.1566021

**Published:** 2025-05-23

**Authors:** Kei Sato, Yuki Yoshida, Shohei Yokota, Hiroki Matsushita, Hidetaka Morita, Masafumi Fukumitsu, Takuya Nishikawa, Kazunori Uemura, Toru Kawada, Keita Saku

**Affiliations:** ^1^ Department of Cardiovascular Dynamics, National Cerebral and Cardiovascular Center Research Institute, Suita, Japan; ^2^ Department of Research Promotion and Management, National Cerebral and Cardiovascular Center, Suita, Japan; ^3^ Bio Digital Twin Center, National Cerebral and Cardiovascular Center, Suita, Japan

**Keywords:** speckle-tracking echocardiography, left ventricular stroke work, myocardial oxygen consumption, left ventricular assist device, left ventricular unloading, coronary flow regulation

## Abstract

**Introduction:**

Assessment of native cardiac function is vital in patients with cardiogenic shock supported by a left ventricular assist device (LVAD), as it is directly related to the critical decision of LVAD management. Left ventricular stroke work (LVSW) can be useful for cardiac assessment to predict survival in cardiogenic shock patients; however, this measurement cannot necessarily be obtained under LVAD support, especially in cases where the aortic valve is closed (i.e., total support). Therefore, we propose a novel echocardiographic parameter named the pressure-strain product (PSP), the product of left ventricular (LV) pressure and LV myocardial strain, as this measurement can be calculated even under LV total support. This study aimed to investigate whether PSP was correlated with pressure-volume (PV) loop-based LVSW and myocardial oxygen consumption under LVAD support.

**Method:**

We used 15 adult goats. An LVAD system was established during open chest surgery by draining blood from the left ventricle and returning it to the carotid artery. LV PV loops were analyzed by measuring LV pressure and volume using sonomicrometry. PV loop-based LVSW was defined as the area surrounded by PV loops. The PSP was defined as the product of the peak LV pressure and global circumferential strain (GCS) using speckle-tracking echocardiography. LVAD support levels were divided into three groups: control, and partial (with residual native cardiac output) and total (without native cardiac output) support. Myocardial oxygen consumption was measured using coronary flow and blood gas analyses. The correlation coefficient was measured using linear regression analysis.

**Results:**

According to each LVAD support level at control, partial support, and total support, LVSW was 1,748 ± 867, 840 ± 467, and 290 ± 262 mmHg·ml, while PSP was 2,341 ± 507, 1,836 ± 768, and 539 ± 269 mmHg·%, respectively. PSP (*r* = 0.54) showed the strongest correlation with PV loop-based LVSW among other echocardiographic parameters, including LV end-diastolic volume (*r* = 0.37), GCS (*r* = 0.40), and echo-based LVSW (*r* = 0.50). PSP level was significantly associated with myocardial oxygen consumption (*r* = 0.55).

**Conclusion:**

PSP significantly correlated with PV loop-based LVSW at various LVAD support levels. PSP can be a non-invasive parameter for assessing myocardial metabolism under LVAD support, potentially reflecting myocardial oxygen consumption.

## Introduction

For patients with cardiogenic shock, assessment of cardiac function is imperative to judge the native heart recovery. A percutaneous left ventricular (LV) unloading device (i.e., Impella) can decrease LV end-diastolic pressure and volume, which reduces the myocardial workload and oxygen demand of the LV myocardium (1, 2). This LV unloading can contribute to myocardial protection and recovery (3); thus, accurate assessment of LV workload has been gaining increasing attention among clinicians (4). However, there is a knowledge gap regarding the accurate measurement of LV workload under device support. In particular, this is challenging with total LV support where the aortic valve constantly closes without LV ejection. Under these conditions, conventional parameters such as cardiac power output cannot be calculated. Therefore, a substitute is needed to evaluate the cardiac workload, indicating LV protection and recovery under LV unloading.

Echocardiographic myocardial strain captures myocardial deformation, indicating cardiac contractility. This measurement can sensitively detect subtle changes in cardiac function and can be useful even with mechanical circulatory support (5). Furthermore, the product of echocardiographic strain and blood pressure, named the pressure-strain product (PSP), has been reported to correlate with LV stroke work (LVSW) in a few animal models (6–8). Considering that LVSW can predict survival better than conventional cardiac parameters, including LV ejection fraction, in the cardiac intensive care unit (9), it is worth investigating whether PSP can estimate LVSW reflecting myocardial oxygen consumption under left ventricular assist device (LVAD) support.

This study aimed to evaluate whether PSP can be correlated with pressure-volume (PV) loop-based LVSW using a large animal model supported by an open-chest LVAD system across various LVAD support levels. Additionally, the relationship of PSP with myocardial oxygen consumption and coronary perfusion were investigated to evaluate LV protection.

## Materials and methods

### Ethics for animal experiments

Animal care strictly adhered to the Guiding Principles for the Care and Use of Animals in the Field of Physiological Sciences, approved by the Physiological Society of Japan. All the experiments were approved by the Animal Subjects Committee of the National Cerebral and Cardiovascular Center.

### Experimental preparation

We used 15 adult goats (Saanen breed of *Capra aegagrus hircus*), weighing 36–54 kg, with a sex distribution of three males and twelve females. Anesthesia was induced using intramuscular xylazine (2 mg/kg), pentazocine (15 mg), and midazolam (10 mg), followed by endotracheal intubation. A laryngotracheal separation was performed to avoid aspiration pneumonia. Separate ventilation for each lung was applied using a double-lumen endotracheal tube to apply different levels of positive end-expiratory pressure (i.e., 15 mmHg for the right lung and 10 mmHg for the left lung). This procedure prevented severe atelectasis of the right lung during left thoracotomy in the right lateral position. Eight and 10 Fr sheaths were inserted into the bilateral femoral artery and vein, respectively (one artery for the measurement of arterial pressure, another artery for collecting blood samples, one vein for the fluid line, and another vein for continuous infusion of muscle relaxant). A 10 Fr long sheath was inserted into the right carotid vein to access the right atrium for a 6 Fr catheter to measure coronary sinus blood gas. Heparin (100 IU/kg) was prophylactically administered to avoid catheter thrombosis and subsequent pulmonary embolism. A 5 Fr pressure catheter (Millar Instruments, Houston, TX, USA) was inserted through the 8 Fr femoral artery catheter into the ascending aorta to measure the aortic pressure.

### Sedation and ventilation settings

An appropriate level of anesthesia was maintained by continuous inhalation of sevoflurane (1%–2%) and rocuronium (10 mg/h) drop infusion. Additional intravenous fentanyl and propofol were administered when required. Ventilation was performed using volume control with a fraction of inspired oxygen ranging from 0.6 to 1.0 to maintain PaO_2_ >80 mmHg. The tidal volume was set at 8–10 ml/kg and the respiratory rate was adjusted to 12–20 breaths per minute to maintain PaCO_2_ at approximately 40 mmHg.

### Open chest surgery

Left thoracotomy was performed in the left fourth intercostal space in the right lateral position. Following the cut of the pericardium, four ultrasound crystals for sonomicrometry (Sonometrics Corporation, London, Ontario, Canada) were inserted into the myocardium and secured with a tourniquet: one pair at the base and apex of the LV wall and another pair at the middle part of the posterior and anterior LV wall. A 5 Fr pressure catheter (Millar Instruments, Houston, TX, USA) was inserted into the LV cavity from the LV free wall to continuously monitor the LV pressure. A 6 Fr catheter (Judkins Right 3.5, Medtronic, Minneapolis, MN, USA) was inserted into the coronary sinus to measure blood gas. Three (3PSB2171, Transonic Systems, Ithaca, NY) and 20 mm flow probes (20PAX1232, Transonic Systems) were attached to the left main trunk and pulmonary artery to evaluate coronary artery flow and pulmonary artery flow, respectively. The stroke volume measured using the pulmonary artery flow probe was used to correct the volume measured using sonomicrometry.

### LVAD system

Following heparinization with 300 IU/kg, a 25 Fr drainage cannula and 19 Fr returning cannula were inserted into LV and left carotid artery, respectively, with the support of fluoroscopy. The tip of the return cannula was placed into the aortic arch. A centrifugal pump (Senko Medical Instrument Mfg. Co., Tokyo, Japan) was used for the LVAD support. Partial support was defined as the maximum support that allowed residual native cardiac output, whereas total support was defined as the support that eliminated native cardiac output.

### Echocardiography

Epicardial echocardiography was performed using an E95 ultrasound machine (GE Healthcare, Chicago, IL, USA) with a 6Sc echo probe. All images were transferred to a separate workstation and analyzed offline by an experienced cardiologist using the Echopac software (GE Healthcare, Chicago, IL, USA). A speckle-tracking echocardiography parameter, the global circumferential strain (GCS) at the LV mid-papillary level, was calculated, and automated measurement was applied to the appropriate echo loops. PSP was calculated as the product of GCS (absolute value) and peak LV pressure (Supplementary Figure S1). The appropriate image quality was selected when the software approved the quality of the speckle tracking as well as a visual assessment by an investigator. End-diastolic and end-systolic timing markers were manually adjusted, if required. A frame rate of 43/s was applied for strain assessment.

### Measurement of LVSW and pressure-volume area using PV loop

LVSW, the area enclosed by the PV loop, was calculated using Green's theorem and the formula embedded in the LabChart 8 software (AD Instruments, Dunedin, New Zealand) (6):

$$\overset{b}{\underset{i\;=\;a}{\sum }}\begin{array}{c} \left\{\left(v_{i}p_{i\;+\;1})\;-\;(v_{i\;+\;1}p_{i}\right)\right\} \end{array}$$

where a and b denote the start and end positions of the loop, respectively. The loop is “closed” by setting v_i_ _+_ _1_ and p_i_ _+_ _1_ to v_a_ and p_a_ when i = b.

Multiple PV loops were generated by altering the preload through inferior vena cava balloon occlusion, and the end-systolic pressure-volume relationship (ESPVR), end-diastolic pressure-volume relationship (EDPVR), and V_0_ (i.e., the intersection of ESPVR with the LV volume axis) were calculated. The area enclosed by the ESPVR, EDPVR, and PV loop was defined as the pressure-volume area (PVA). The PVA was measured using an in-house program (MATLAB R2018b; Mathworks, Natick, MA, USA).

### Measurement of myocardial oxygen consumption and coronary vascular resistance

Myocardial oxygen consumption (MVO_2_) was measured using the following formula:

$$\begin{array}{rl} \mathrm{MV}O_{2} & =\{1.36\times \mathrm{Hb}\times (S_{a}O_{2}-S_{\mathrm{cs}}O_{2})\;\times (\mathrm{coronary}\;\mathrm{flow})\}/\\ & \{\left(\mathrm{heart}\;\mathrm{rate})\;\times \mathrm{LV}\;\mathrm{weight}\;(\mathrm{per}\;100\;\mathrm{mg})\right\} \end{array}$$

where Hb is the hemoglobin concentration (g/dl) and S_a_O_2_ and S_cs_O_2_ are the oxygen saturations of the artery and coronary sinus, respectively. Coronary vascular resistance (CVR) was calculated using the following formula:

CVR=CPP/(coronaryflow)

where CPP is coronary perfusion pressure defined as MAP-mean LV pressure.

### Experimental protocol

**Figure 1:**
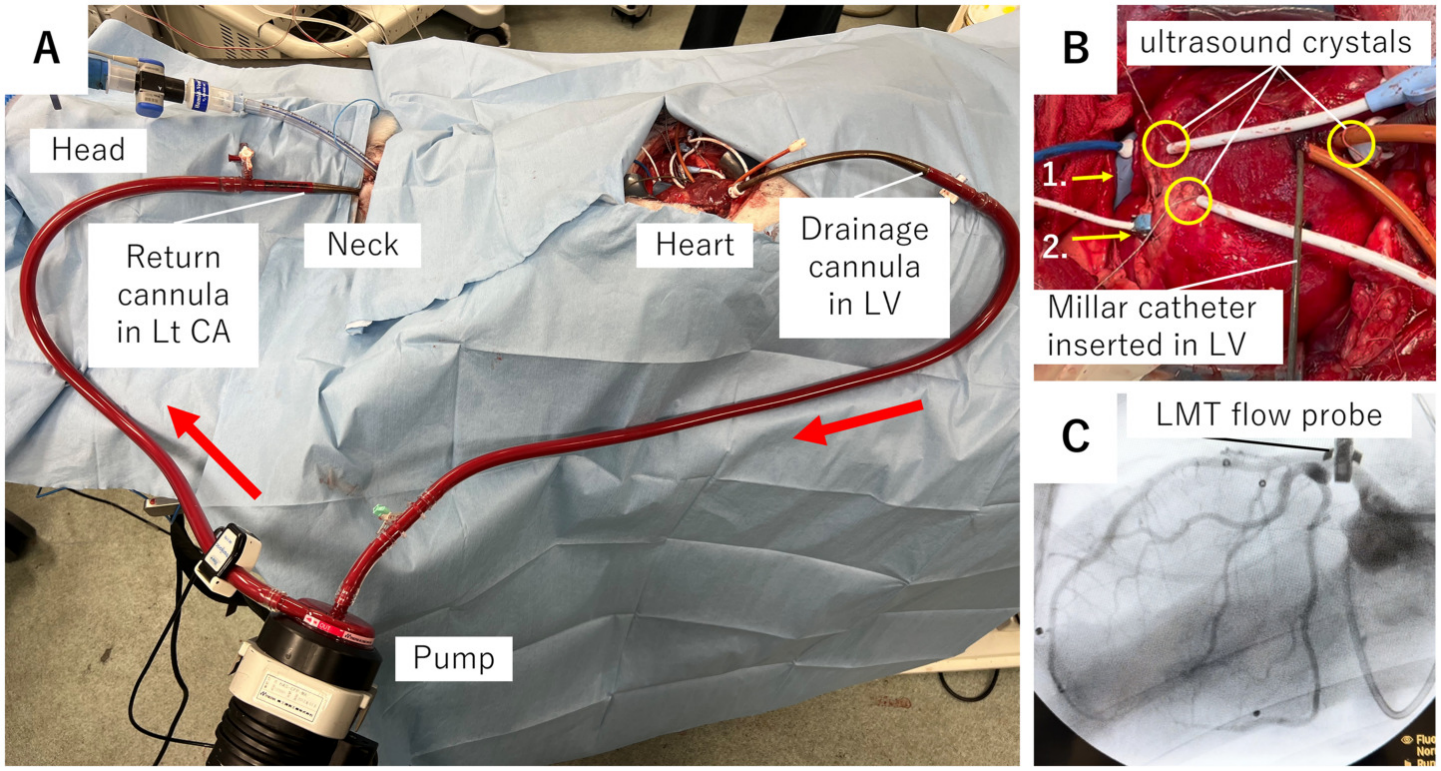
Overview of experimental setup. **(A)** Picture describing LVAD system. Blood sucked from a drainage cannula at the left ventricle flows through a pump to the carotid artery via the return cannula. Red arrows show the direction of blood flow. **(B)** Picture showing the location of ultrasound crystals and flow probe. (1) A flow probe at pulmonary artery; (2) a flow probe at left main trunk of coronary artery. **(C)** Cine film showing the left coronary arteries and the flow probe at the left main trunk. CA, carotid artery; LMT, left main trunk; Lt, left; LVAD, left ventricular assist device.

**Figure 2:**
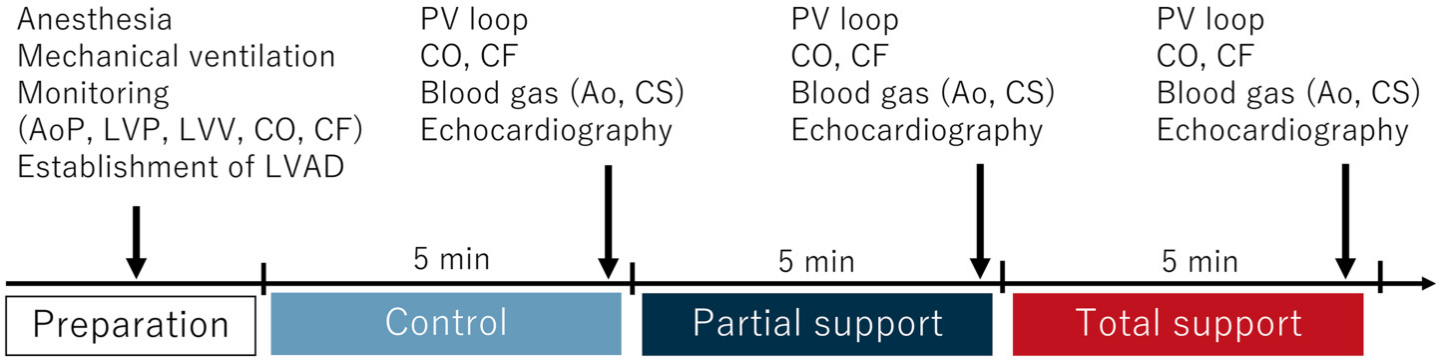
Study protocol. Following the preparation, PV loop, hemodynamic parameters, blood gas, and echocardiographic parameters were assessed after 5 min stabilization at control, partial, and total support of LVAD. Ao, aorta; AoP, aortic pressure; CF, coronary flow; CO, cardiac output; CS, coronary sinus; LVAD, left ventricular assist device; LVP, left ventricular pressure; LVV, left ventricular volume; PV, pressure-volume.

Following experimental preparation (Figure 1), hemodynamic parameters (i.e., LV pressure and PV loop-based LVSW), blood gas (i.e., one from the femoral artery and another from the coronary sinus), and an echocardiographic image of the LV short-axis view were assessed after 5 min stabilization at the control and partial and total support of the LVAD (Figure 2).

### Statistics

Friedman test was conducted to compare the hemodynamic or echocardiographic parameters during the three different LVAD support levels. The Bonferroni *post hoc* test was applied for inter-group comparisons. The correlation coefficient was measured using linear regression analysis. *p* < 0.05 was considered statistically significant. Statistical tests were conducted using EZR (version 1.68) (10) in R Commander (version 2.9-1) and Microsoft Excel (Microsoft Corp., Redmond, WA, USA).

## Results

### Representative hemodynamic waveforms based on LVAD support levels

**Figure 3:**
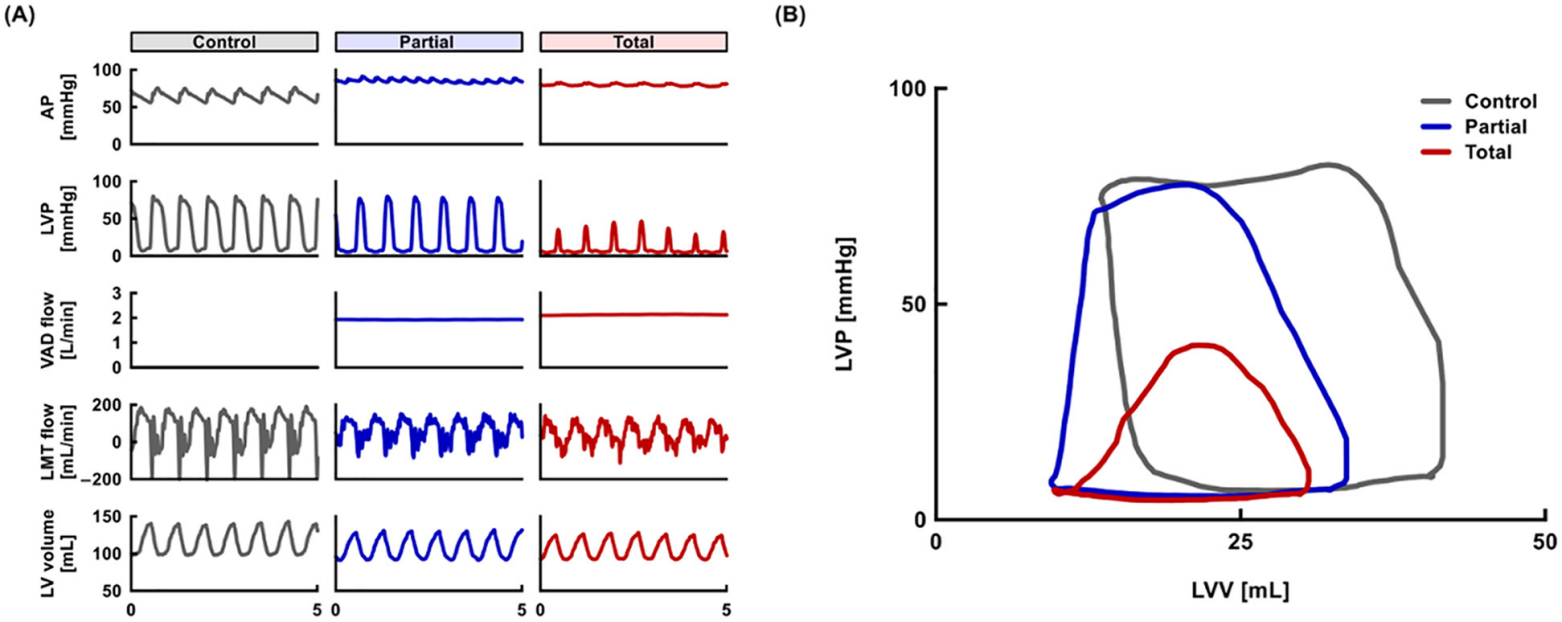
Representative hemodynamics. **(A)** Wave forms of AP, LVP, VAD flow, LMT flow, and LV volume at control, partial, and total support. **(B)** Pressure-volume loops at control, partial, and total support. AP, aortic pressure; LMT, left main trunk; LV, left ventricular; LVP, left ventricular pressure; LVV, left ventricular volume; VAD, ventricular assist device.

Baseline characteristics of animals are described in the Table 1. Representative hemodynamic waveforms are shown in Figure 3. As the LVAD support level increased, LV pressure and volume decreased and the PV loop shifted left and downward. In addition, as the aortic pressure gradually increased, the coronary flow in the left main trunk showed a decreasing trend.

**Table 1:** Baseline characteristics including hemodynamics and echocardiographic parameters.

Parameters	Median (IQR)
BW (kg)	47 (45–50)
HR (/min)	79 (68–97)
MAP (mmHg)	73 (58–77)
Peak LVP (mmHg)	87 (70–92)
LVEDP (mmHg)	11 (10–13)
Hb (g/dl)	9.4 (8.5–10.5)
SaO_2_ (%)	99 (98–100)
ScsO_2_ (%)	52 (49–69)
LVEDV (ml)	63 (53–73)
LVESV (ml)	21 (15–29)
LVSV (ml)	40 (34–44)
LVEF (%)	68 (61–71)
GCS (absolute) (%)	27.6 (26.5–31.7)
PSP (mmHg·%)	2,427 (2,252–2,699)
LVSW (based on PV-loop) (mmHg·ml)	1,728 (1,052–2,183)
LV mass (g)	115 (110–119)

BW, body weight; GCS, global circumferential strain; Hb, hemoglobin; HR, heart rate; IQR, interquartile range; LVEDP, left ventricular end-diastolic pressure; LVEDV, left ventricular end-diastolic volume; LVEF, left ventricular ejection fraction; LVESV, left ventricular end-systolic volume; LVP, left ventricular pressure; LVSV, left ventricular stroke volume; LVSW, left ventricular stroke work; MAP, mean arterial pressure; PSP, pressure-strain product; SaO2, arterial oxygen saturation; ScsO2, coronary sinus oxygen saturation.

### Representative echocardiographic images and PSP based on LVAD support levels

**Figure 4:**
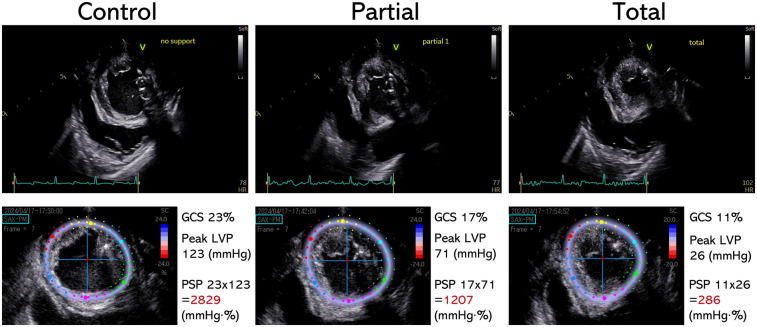
Representative echocardiographic images and PSP calculation. Upper pictures: Echocardiographic images of left ventricular short axis view at the mid-papillary level. Lower pictures: Speckle-tracking echocardiographic analysis corresponding to the upper pictures. PSP at each LVAD support level is shown as the product of GCS and peak LV pressure. GCS, global circumferential strain; LVAD, left ventricular assist device; LVP, left ventricular pressure; PSP, pressure-strain product.

Representative ultrasound images (LV short axis view) and speckle-tracking analyses are shown in Figure 4. As LVAD support level increased, GCS, LV systolic pressure, and subsequent PSP decreased.

### Trends in hemodynamic and echocardiographic parameters based on LVAD support levels

**Figure 5:**
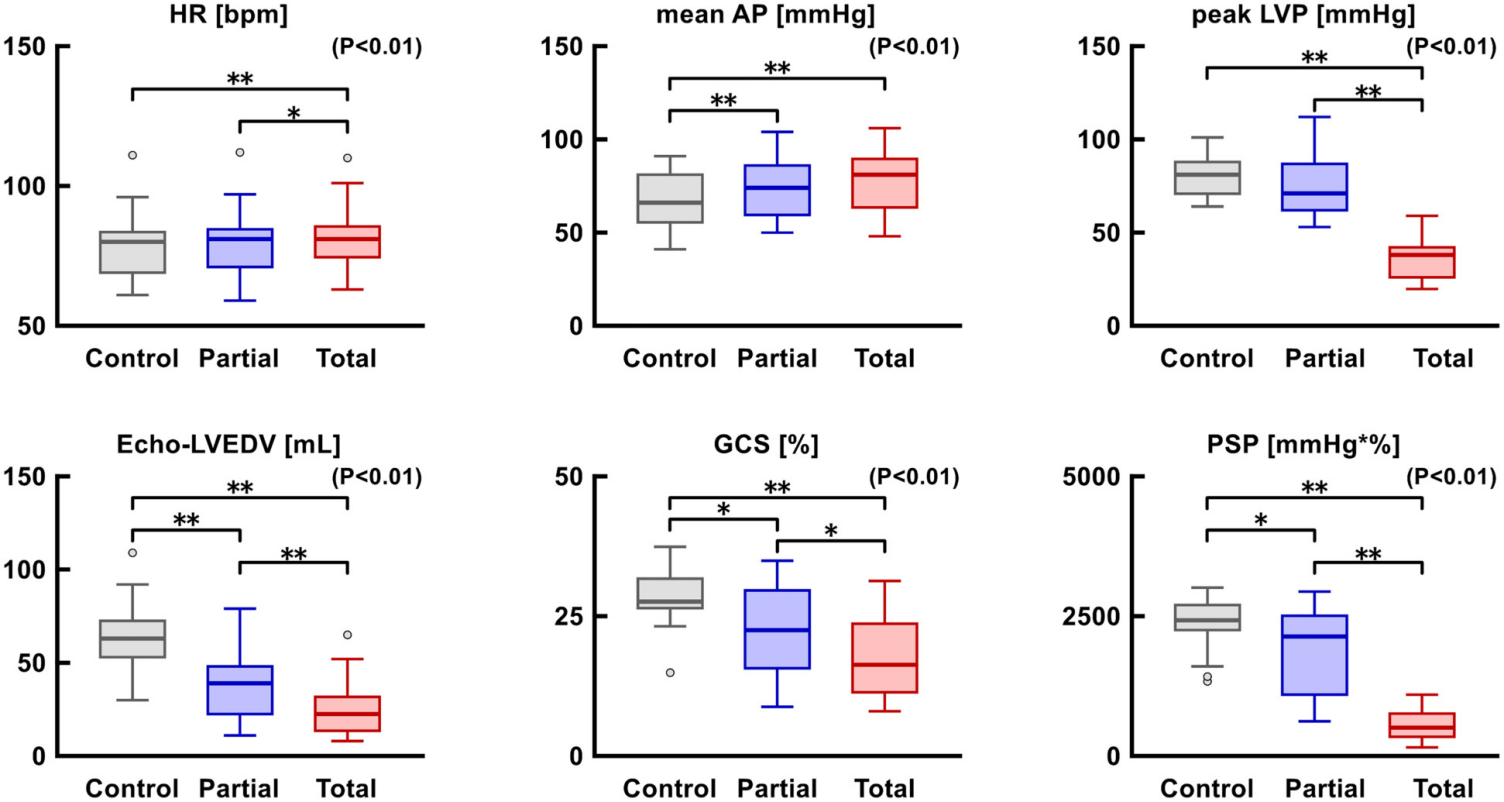
Trends in hemodynamic (upper row) and echocardiographic (lower row) parameters. *p < 0.05, **p < 0.01. AP, arterial pressure; GCS, global circumferential strain; HR, heart rate; LVEDV, left ventricular end-diastolic volume; LVP, left ventricular pressure; PSP, pressure-strain product.

Hemodynamic parameters, including heart rate, mean arterial pressure, and LV systolic pressure, are shown in Figure 5. As LVAD support levels increased, the heart rate and mean arterial pressure increased significantly, whereas LV systolic pressure decreased significantly. Similarly, the LV end-diastolic volume (EDV), GCS, and PSP significantly declined when LVAD support increased.

### Correlation analysis between PV loop-based LVSW and echo/hemodynamic parameters

**Figure 6:**
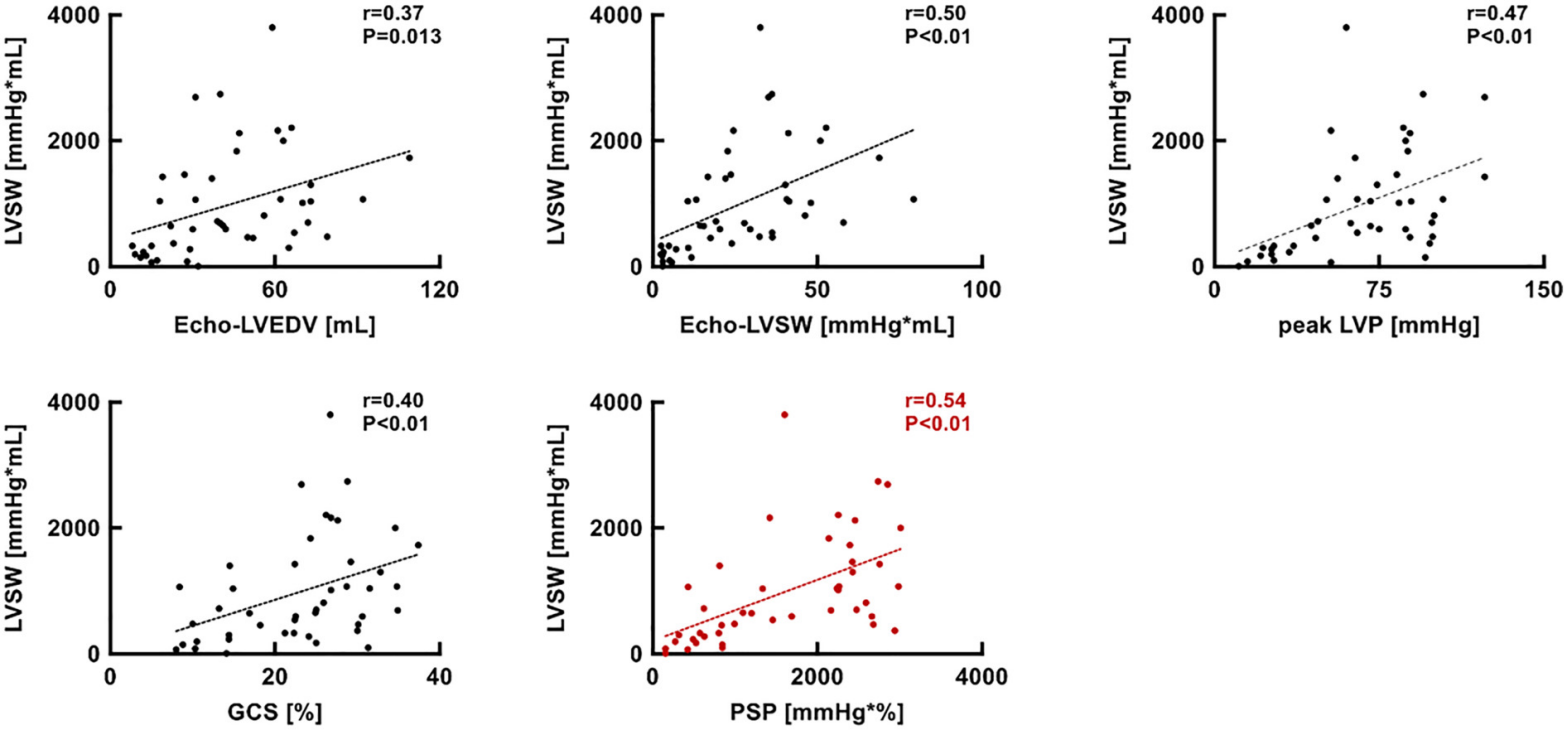
Scatter plots showing the relationship between PV loop-based LVSW and echo-LVEDV, echo-LVSW, peak LVP, GCS, and PSP. GCS, global circumferential strain; LVEDV, left ventricular end-diastolic volume; LVP, left ventricular pressure; LVSW, left ventricular stroke work; PSP, pressure-strain product; PV, pressure-volume.

Scatter plots of PV loop-based LVSW vs. echo-based LV EDV, echo-based LVSW, GCS, and PSP are shown in Figure 6. PSP showed the strongest correlation (*r* = 0.54, *p* < 0.01) with the PV loop-based LVSW among other parameters.

### Trends in cardiac energetics and coronary perfusion based on LVAD support levels

**Figure 7:**
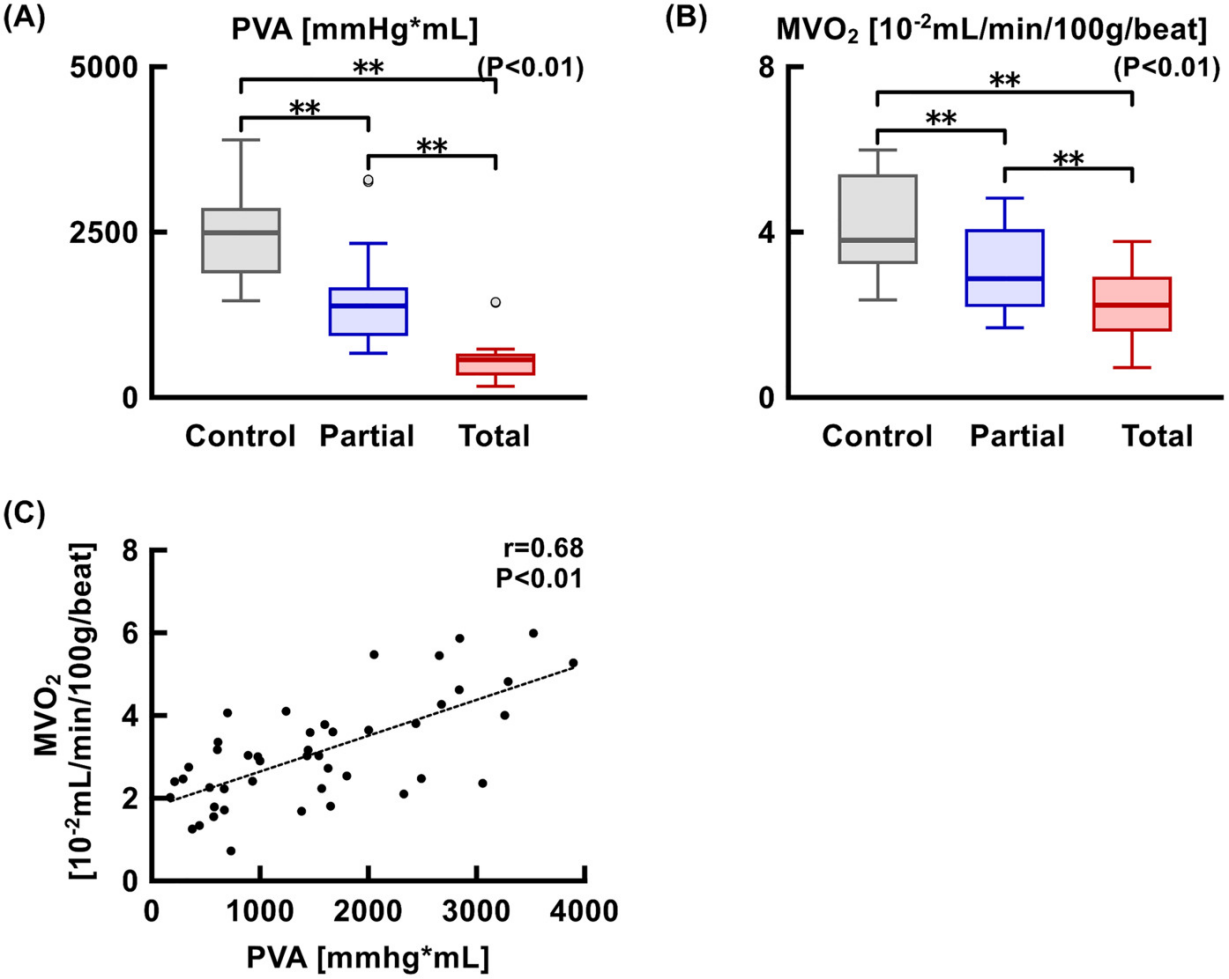
Trends in PVA **(A)** and MVO_2_ **(B)** at each LVAD support level and the correlation between the two parameters **(C)** MVO_2_, myocardial oxygen consumption; PVA, pressure-volume area.

**Figure 8:**
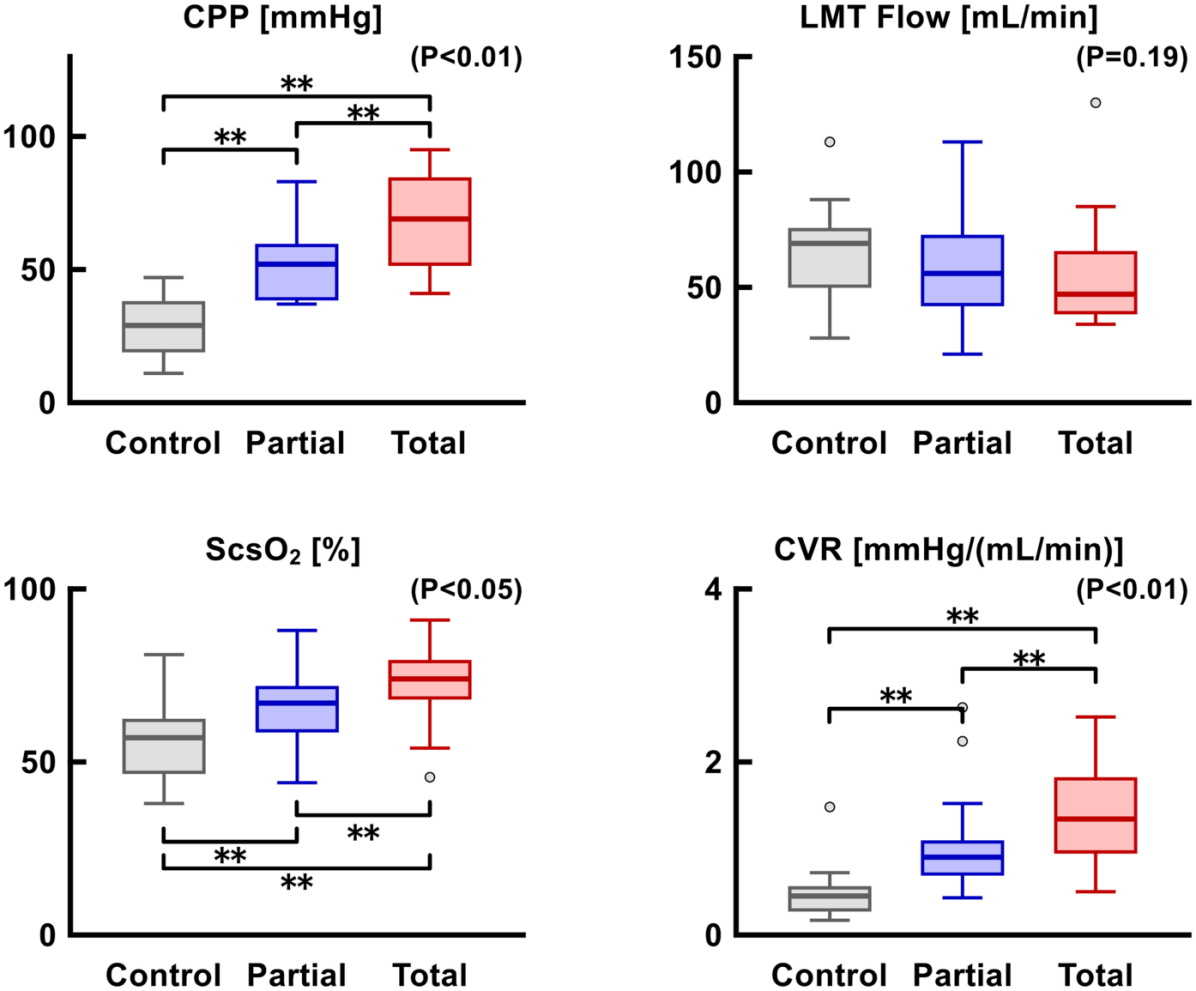
Trends in CPP, LMT flow, S_CS_O_2_, and CVR according to LVAD support. CPP, coronary perfusion pressure; CVR, coronary vascular resistance; LMT, left main trunk; ScsO_2_, oxygen saturation of coronary sinus blood.

MVO_2_ and PVA were significantly reduced as LVAD support level increased (Figure 7). MVO_2_ and PVA were significantly correlated (*r* = 0.68, *p* < 0.01). Coronary perfusion pressure, S_CS_O_2_, and coronary vascular resistance significantly increased, whereas coronary flow at the left main trunk showed a decreasing trend as LVAD support increased (Figure 8).

### Correlation analysis of PSP with both cardiac energetics and coronary perfusion

**Figure 9:**
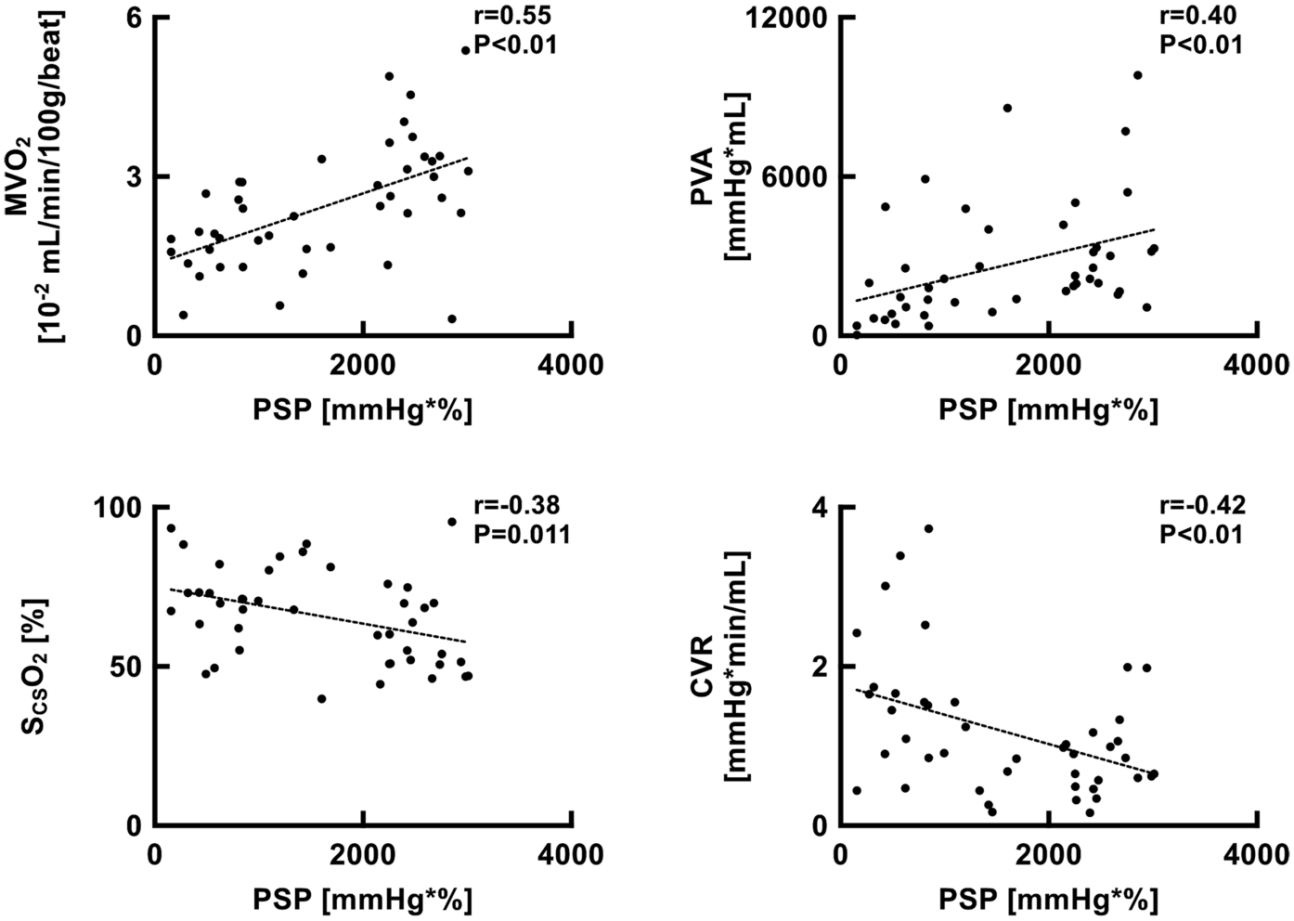
Scatter plots showing the relationship between PSP and MVO_2_, PVA, S_CS_O_2_, and CVR. CVR, coronary vascular resistance; MVO_2_, myocardial oxygen consumption; PSP, pressure-strain product; PVA, pressure-volume area; ScsO_2_, oxygen saturation of coronary sinus blood.

Pressure-strain product were significantly correlated with LV MVO_2_ (*r* = 0.55, *p* < 0.01), PVA (*r* = 0.40, *p* < 0.01), S_CS_O_2_ (*r* = 0.38, *p* = 0.011), and coronary vascular resistance (CVR) (*r* = 0.42, *p* < 0.01) (Figure 9).

## Discussion

Pressure-strain product showed the strongest correlation with PV loop-based LVSW among other echocardiographic parameters, including echo-based LV EDV, echo-based LVSW, and GCS. PSP is also related to myocardial oxygen consumption, which can be a reasonable parameter reflecting LV unloading and protection.

### Rationale of correlation between PSP and LVSW

Pressure-strain product was conceptualized using a pressure-strain loop, where the volume was replaced with the myocardial strain in the pressure-volume loop (Supplementary Figure S1) (6–8). Russell et al. reported that the area surrounded by the LV pressure-strain loop, called myocardial work, was significantly correlated with PV loop-based LVSW (11). In addition, regional myocardial work could reflect myocardial glucose metabolism, as measured by positron emission tomography. While myocardial work requires vendor-dependent software and uses global longitudinal strain (GLS), we propose a vendor-independent PSP that can be calculated as the product of mean arterial pressure and GCS. Previous studies have shown that PSP calculated using mean arterial pressure (MAP) correlates well with LVSW in various settings, including the ovine model of septic cardiomyopathy (8), brain stem death (7) and cardiogenic shock supported by V-A ECMO (6). The crucial difference between previous studies and the present study is that we examined whether PSP could be measured even under total LV unloading, where significant gap exists between MAP and LVSP. Therefore, we redefined PSP from MAP × GCS as used in previous reports, to peak LVP × GCS to appropriately express LV workload during total LV unloading. Thus, the novelty of our study lies in measuring PSP using a different definition from previous reports and analyzing its correlation with LVSW.

### Potential benefits to use GCS rather than stroke volume or global longitudinal strain in estimating LVSW

To obtain the value of LVSW, measurement of the stroke volume (SV) is essential. As an invasive method, the Swan-Gantz catheter can accurately measure SV as the gold standard; however, this method is not routinely recommended in clinical settings because of its invasive nature and subsequent complications (12). Echocardiography can be applied as a non-invasive method to evaluate SV; however, significant pitfalls regarding variations among sonographers have been reported (13). Speckle-tracking echocardiography-based myocardial work can overcome this limitation by applying automated methods to evaluate myocardial strain, achieving high reproducibility (14). The limitation of this method is the difficulty in obtaining three appropriate echo images (2-, 3-, and 4-chamber views from the LV apex) to calculate GLS (14), especially in ICU settings where patients cannot cooperate with echo sonographers to adjust respiratory timing. In contrast, GCS can be obtained using the LV short axis view alone, which is simpler than obtaining the GLS. Therefore, PSP consisting of GCS can be a user-friendly measurement for clinicians to estimate LVSW.

### The significance of PSP for the cardiac assessment and the optimization of LVAD support

Left ventricular stroke work was calculated using echocardiography as follows: LVSV × MAP × 0.0134 (9). However, the LVSV does not exist under the condition of total LV support, where the aortic valve constantly closes. In this situation, we cannot measure LVSV and subsequent LVSW using the LV outflow tract velocity-time integral, which is one of the most common parameters in clinical settings. Although the value of LVSV, defined as the gap between LV EDV and end-systolic volume, can be obtained under LV total support, the LVSW estimated using this method was less correlated with PV loop-based LVSW than PSP (Figure 6). Given that LV myocardial strain can be obtained even under the condition of aortic valve closure, PSP has an advantage over echo-based LVSW and potentially evaluates the LV workload in patients with cardiogenic shock, even under LV total support.

With the emergence of percutaneous LVAD (i.e., Impella®), clinicians need to consider LV workload and myocardial oxygen metabolism as well as hemodynamic stability to optimally utilize the device. Considering that PSP was significantly correlated with MVO_2_ and PVA (Figure 9), PSP can be utilized for the LVAD optimization by potentially reflecting myocardial oxygen metabolism. In the present study, although coronary perfusion pressure increased with higher levels of LVAD support, coronary flow remained limited, and coronary vascular resistance was elevated (Figure 8). This may be attributed to the reduced myocardial oxygen demand under LVAD support, which limits coronary flow by increasing coronary vascular resistance. This phenomenon has also been reported in a previous study (15), suggesting a mechanism of optimization known as coronary vascular autoregulation.

### Validity of the model

The present study aimed to evaluate the physiological relationships between the pressure-strain product (PSP) and myocardial oxygen consumption. This objective necessitated highly invasive procedures, including the implantation of sonomicrometry probes within the left ventricular myocardium under open-chest conditions, placement of a flow probe around the coronary artery, and insertion of a catheter into the coronary sinus. Considering the experimental duration and procedural success rate, a normal heart model was employed. Further investigation using an appropriate model is required to determine whether the present findings are applicable to failing hearts. Nevertheless, PSP may represent a valuable tool for assessing native cardiac function during LVAD weaning in the recovery phase.

### A future perspective

Because the current Impella (e.g., Impella 5.5) has a system called Smart Assist, which is equipped with a catheter to estimate LV pressure, our method can be immediately applied in clinical settings to evaluate LVSW without additional catheters. Considering that a centrifugal pump rather than an Impella was used under normal heart model in this study, we are currently conducting experiments using an Impella 5.5 in a failed heart model to investigate whether the results observed in the current study can be applicable. Future clinical studies are required to investigate whether PSP can be useful for optimizing Impella support level and to clarify the optimal threshold of PSP to predict cardiac recovery.

### Limitations

Our study had a few limitations. First, we applied epicardial echocardiography, which is different from clinical practice. Pressure from the echo probe might affect echocardiographic parameters, including the GCS. Second, peak LV pressure, rather than mean arterial pressure, was used in this study for the calculation of PSP, which might have overestimated the LVSW. However, considering the significant gap between MAP and peak LV pressure in LVAD total support, the method applied in this study is reasonable compared to the previously reported method using MAP. Third, since hemodynamic assessments were performed following 5-min stabilization after changing the LVAD flow settings, longer-term effects were not considered. Finally, the model of normal hearts rather than deteriorated hearts was used in the current study. In the condition of cardiogenic shock, LV contractility is reduced, LV afterload is increased, and LV preload is elevated compared to a normal heart. These conditions can affect LV strain, LV pressure and subsequent PSP. In addition, the impact of coronary vascular autoregulation on the results may differ in failed hearts. Therefore, future studies applying a cardiogenic shock model are necessary to validate our findings under these distinct physiological conditions.

## Conclusions

Echocardiography-based PSP significantly correlated with PV loop-based LVSW under various LVAD support levels, including total LV support. PSP can be useful in the noninvasive monitoring of myocardial metabolism by potentially reflecting myocardial oxygen consumption. Future clinical studies are required to investigate whether PSP can contribute to the optimization of Impella support and the prediction of cardiac recovery using the Impella device.

## Data Availability

The original contributions presented in the study are included in the article/Supplementary Material, further inquiries can be directed to the corresponding authors.

## Supplementary material

Supplementary Figure S1 Schematic of PSP. PSP was measured as the area of a rectangle consisting of the LV pressure and LV strain. The PSP of control (light blue), partial support (dark blue), and total support (red) are illustrated based on each LV systolic pressure and global circumferential strain. PSP, pressure-strain product.
