# Supplementary figures and images for: Pressure-strain product reflects left ventricular stroke work under a wide range of left ventricular assist device support levels
Source: Front Cardiovasc Med. 2025 May 23;12:1566021. doi: 10.3389/fcvm.2025.1566021 (PMC12141206; doi:10.3389/fcvm.2025.1566021)

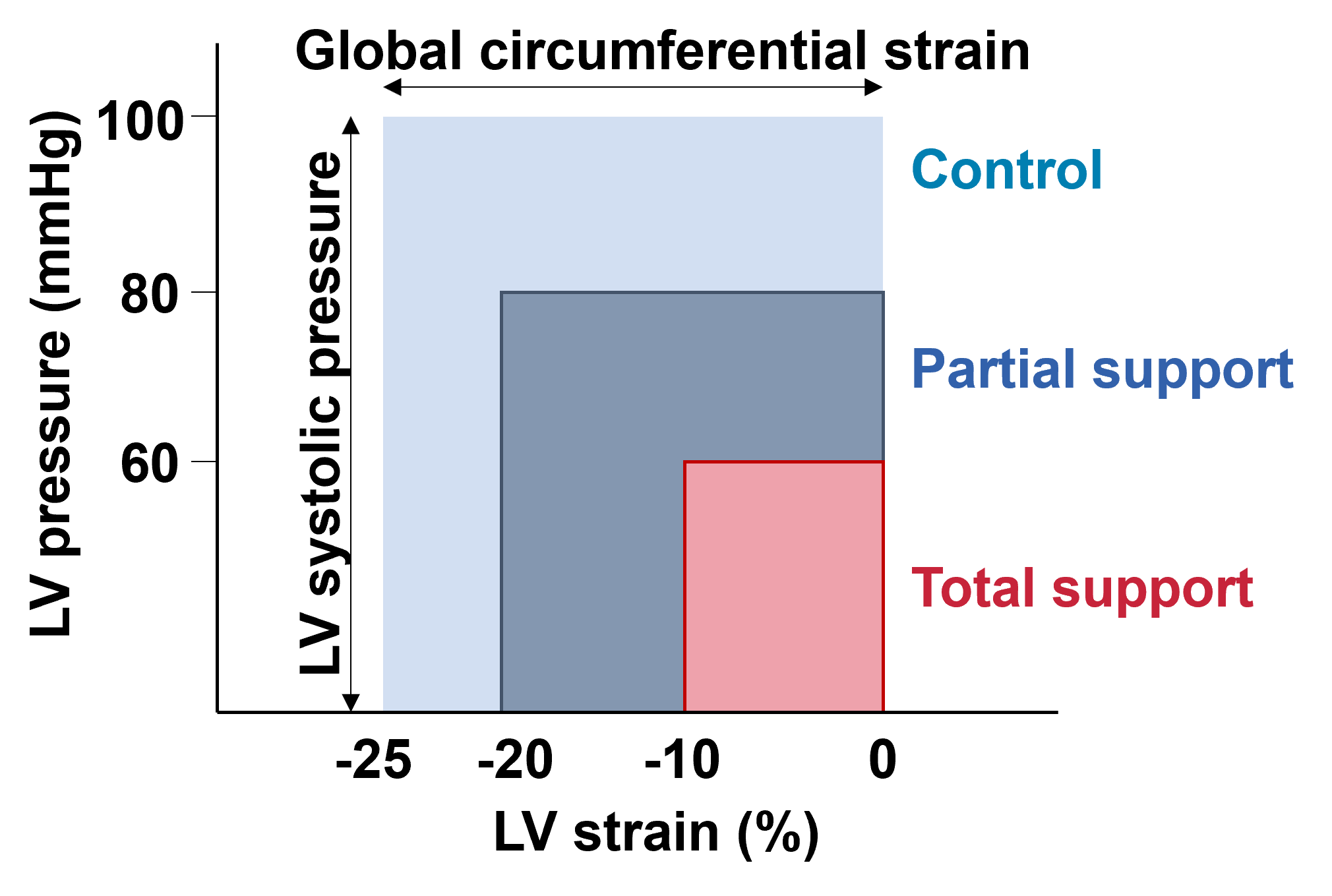

Supplement: Supplementary Figure S1 — Schematic of PSP. PSP was measured as the area of a rectangle consisting of the LV pressure and LV strain. The PSP of control (light blue), partial support (dark blue), and total support (red) are illustrated based on each LV systolic pressure and global circumferential strain. PSP, pressure-strain product. [file Image1.tif]
